# Circulating ANGPTL8/Betatrophin Is Increased in Obesity and Reduced after Exercise Training

**DOI:** 10.1371/journal.pone.0147367

**Published:** 2016-01-19

**Authors:** Mohamed Abu-Farha, Devarajan Sriraman, Preethi Cherian, Irina AlKhairi, Naser Elkum, Kazem Behbehani, Jehad Abubaker

**Affiliations:** 1 Biochemistry and Molecular Biology Unit, Dasman Diabetes Institute, Kuwait City, Kuwait; 2 Tissue Banking Unit, Dasman Diabetes Institute, Kuwait City, Kuwait; 3 Sidra Medical and Research Center, Doha, Qatar; Communaute d\'Universites et d\'Etablissements Lille Nord de France, FRANCE

## Abstract

**Objective:**

ANGPTL8 is a liver and adipose tissue produced protein that regulates the level of triglyceride in plasma as well as glucose homeostasis. This study was designed to evaluate the level of ANGPTL8 in obese and non-obese subjects before and after exercise training.

**Methods:**

A total of 82 non-obese and 62 adult obese were enrolled in this study. Subjects underwent a three months of exercise training. Both full length and C-terminal 139–198 form of ANGPTL8 were measured by ELISA.

**Results:**

Our data show that the full length ANGPTL8 level was increased in obese subjects (1150.04 ± 108.10 pg/mL) compared to non-obese (775.54 ± 46.12) pg/mL (*p*-Value = 0.002). C-terminal 139–198 form of ANGPTL8 was also increased in obese subjects 0.28 ± 0.04 ng/mL vs 0.20 ± 0.02 ng/mL in non-obese (*p*-value = 0.058). In obese subjects, the levels of both forms were reduced after three months of exercise training; full length was reduced from 1150.04 ± 108.10 pg/mL to 852.04 ± 51.95 pg/mL (*p*-Values 0.015) and c-terminal form was reduced from 0.28 ± 0.04 ng/mL to 0.19 ± 0.03 ng/mL (*p*-Value = 0.058). Interestingly, full length ANGPTL8 was positively associated with fasting blood glucose (FBG) in non-obese (r = 0.317, p-Value = 0.006) and obese subjects (r = 0.346, p-Value = 0.006) C-terminal 139–198 form of ANGPTL8 on the other hand, did not show any correlation in both groups.

**Conclusion:**

In conclusion, our data demonstrate that ANGPTL8 was increased in obesity and reduced after exercise training supporting the potential therapeutic benefit of reducing ANGPTL8. The various forms of ANGPTL8 associated differently with FBG suggesting that they have different roles in glucose homeostasis.

## Introduction

Type 2 Diabetes (T2D) is caused by the inability of β-cells to compensate for insulin resistance leading to uncontrolled hyperglycemia [1,2,3]. Stimulating β-cells proliferation has been suggested as an ultimate treatment for T2D that can lead to better control of blood glucose [4]. The large increase in β-cell proliferation induced by a new hormone called betatrophin (ANGPTL8) in response to insulin resistance has attracted tremendous attention in the field [5,6,7]. Betatrophin is mainly expressed in the liver and adipose tissue and secreted into circulation [5,8,9,10,11]. Its ability to induce over 17-fold increase in β-cell proliferation in response to insulin resistance suggested that betatrophin can be used to augment other T2D treatments and reduce dependency on insulin injections [5,6,7]. However, recent data by Gusarova et al., has challenged this claim showing that β-cell proliferation in mice lacking ANGPTL8 was not affected under insulin resistance [12]. Their claim was also supported in another report published by the original betatrophin group showing that their betatrophin null mice had a normal β-cell proliferation under induced insulin resistance state [13]. This work was also supported by Cox et al., which showed that overexpression of betatrophin does not increase β-cell proliferation in mice [14]. In support of this data, our recent data has shown that increased betatrophin level in T2D subjects was not correlating with glucose level or insulin level [15]. Nonetheless, at the time of preparing this manuscript Chen et al., showed that betatrophin was capable of inducing β-cell regeneration in rats [16].

Recent reports have looked at the expression level of ANGPTL8 in various populations showing conflicting data in regard to its expression level in obesity and diabetes. However, the majority of data showed that ANGPTL8 was increased in type 1 diabetes as well as T2D including our recent data where we used over 1600 subjects to show that ANGPTL8 was increased in T2D [15,17,18,19,20,21,22,23]. Other reports have shown that ANGPTL8 was reduced in diabetic subjects [24]. Obesity has also been shown to increase the level of ANGPTL8 by some studies [21] while others showed a decrease [24]. ELISA kits measuring various forms of ANGPTL8 have been suggested as a possible reason behind this discrepancy [25]. In this study we hypothesized that ANGPTL8 was increased in obese subjects due to its role in regulating TG plasma level and in consequences, exercise training will reduce its level. We also investigated the difference between plasma level of full length and C-terminal 139–198 form of ANGPTL8 in non-obese and obese subjects before and after three months of exercise training.

## Research Design and Methods

### Study population and ethical statement

In the present study, a total of 144 subjects were recruited to undergo three months of exercise training as described previously [26,27]. The recruited subjects were classified according to their Body Mass Index (BMI) into non-obese (19.5 and ≤ BMI < 30 kg/m^2^) and obese (30 ≤ BMI < 40 kg/m^2^). A total of 82 non-obese and 62 adult obese were enrolled in this study. Written informed consent was obtained from all subjects before their participation in the study which was approved by the Review Board of Dasman Diabetes Institute and carried out in line with the guideline ethical declaration of Helsinki. All volunteers were subjected to a pre-screening assessment to determine their eligibility to participate in the study. Glucose and lipid profiles were analyzed for all volunteers. Candidates that followed any exercise training within the last 6 months prior to this study, the morbidly obese (i.e. BMI >40 kg/m^2^) and those with prior major illness were excluded from the study. All selected subjects were non-diabetics. In addition, as previously outlined, eligible candidates should not be taking any medication and/or supplement known to influence the body composition or bone mass [26,27].

### Exercise Program

Exercise training program was described previously [26,27]. Briefly, the exercise program was supervised by the Fitness and Rehabilitation Center (FRC) of Dasman Diabetes Institute. Prior to exercise, participants were examined by CPET (symptom-limited maximal incremental cardiopulmonary exercise test) (COSMED Quark, Italy) using an electromagnetically braked cycle ergometer to determine the maximum heart rate (max HR) as well as the response to aerobic exercise as measured by the maximum oxygen consumption (V_O₂ Max_) for each subject. An exercise session includes 10 minutes warming-up and cooling down steps at 50–60% of max HR, along with 40 minutes of the prescribed exercise program at 65–80% of max HR. Prescribed exercise program involved a combination of both moderate intensity of aerobic (30 min) and resistance training using either a treadmill or cycling (10 min). For the duration of the three months period, participants exercised three to five times per week at the recommended HR range. Strength training was performed two to three times a week according to the program plan. Exercise intensity, duration and blood pressures were recorded for each session. All trainings were supervised by qualified fitness professionals and a consultant respirologist from FRC. To assess the effectiveness of the exercise, the same physical stress and fitness tests were performed for all subjects at the end of the exercise program.

### Blood Collection and Anthropometric and Biochemical Measurements

Venous peripheral blood was collected before starting the exercise and after the three months exercise period. Plasma and serum were prepared using vacutainer EDTA tubes and then aliquoted and stored at -80°C until assayed. Plasma was obtained after centrifugation for 10 minutes at 400g at room temperature. Plasma was then aliquoted into cryogenic tubes and stored at -80°C. Physical and anthropometric measurements including blood pressure, body weight, height, waist circumference (WC) and blood pressure were measured at baseline and after 3 months of exercise.

Blood pressure was measured using an Omron HEM-907XL Digital sphygmomanometer. The average of three readings with 5–10 minutes rest between each reading is reported. Using calibrated electronic weighing scale and inflexible height measuring bars, height and weight were recorded with participants wearing light indoor clothing and barefooted. After a normal exhalation, with arms relaxed at the sides, at the highest point of the iliac crest and at the mid-axillary line, WC was measured using a constant tension tape. The formula used to calculate BMI is the standard BMI formula: body weight (in kilograms)/height (in meters squared).Whole-body composition was determined by dual-energy radiographic absorptiometry device (Lunar DPX, Lunar radiation, Madison, WI). Fasting blood Glucose (FBG) triglyceride (TG), total cholesterol (TC), low density lipoprotein (LDL) and high density lipoprotein (HDL) were measured on the Siemens Dimension RXL chemistry analyzer (Diamond Diagnostics, Holliston, MA). Glycated hemoglobin (HbA1C) was determined using the Variant^TM^ device (BioRad, Hercules, CA).

### ELISA ANGPTL8 and Level

The plasma samples which had been stored in freezer of -80 ^0^C were thawed on ice and centrifuged at 10000g for 5 minutes at 4 ^0^C to remove any debris. Repeated freeze thaw cycles were avoided. Full length level of ANGPTL8 in circulation was measured using ELISA (Wuhan EIAAB Science co) (catalogue number E1164H) as described previously [15,23,28]. This kit was validated using recombinant ANGPTL8 spiked at known concentration into plasma as described previously. A range of ANGPTL8 concentrations were used to spike in plasma at different dilution factors. The assay showed linearity at dilutions ranging from 1:10–1:40. Recovery of the known proteins ranged from 85–109%. No significant cross reactivity with other proteins was observed. Intra-assay coefficients of variation were 1.2% to 3.8%, while the inter-assay coefficients of variation were 6.8% to 10.2%. C-terminal 139–198 form of ANGPTL8 was measured using ELISA kit recognizing ANGPTL8 region spanning from 139 to 198 amino acid Phoenix Pharmaceuticals (catalogue number EK-051-55). No significant cross reactivity with other proteins was observed. Intra, and inter assay coefficients of variation were less than 9.6%.

### Statistical Analysis

Comparisons between non-obese and obese subjects before and after exercise were made by Student’s t-test. Spearman’s correlation coefficients were estimated to determine associations between ANGPTL8 and FBG measurements and biochemical variables; data was adjusted for age gender and BMI. All data are reported as mean ± standard deviation (SD) and ANGPTL8 was reported as mean ± standard error mean (SEM). All statistical assessments were two-sided and considered to be significant when *P-value* < 0.05. All analyses were performed using SAS (version 9.r; SAS Institute, Cary, NC).

## Results

### Clinical characteristics of population at baseline for non-obese and obese subjects

Clinical and biochemical characteristics of the non-obese and obese subjects at baseline are shown in Table 1. A total of 144 subjects including 82 non-obese and 62 obese subjects. Obese subjects were older and had significantly higher BMI, waist/hip ratio, percent body fat, soft lean mass and total body water (p < .05). Heart rate, systolic blood pressure, FBG, HbA1C, TG, TC, HDL, and LDL were also significantly higher in obese subjects (p < .05).

**Table 1:** Characteristics of the non-obese and obese subjects included in this study.

Variable	Non-obese Average ± SD, N = 82	Obese Average ± SD, N = 62	p-value
**Age (years)**	40.44 ± 11.83	47.19 ± 12.69	**0.001**
**BMI (kg/m**^**2**^**)**	24.66 ± 2.86	34.76 ± 3.22	**<0.001**
**Percent Body Fat**	31.58 ± 5.74	38.41 ± 5.76	**<0.001**
**Soft Lean Mass**	42.78 ± 8.75	54.26 ± 11.64	**<0.001**
**Total Body water**	33.59 ± 6.72	42.95 ± 8.93	**<0.001**
**WC (cm)**	85.38 ± 9.56	108.04 ± 10.83	**<0.001**
**Hip (cm)**	103.71 ± 13.48	117.96 ± 8.87	**<0.001**
**Heart Rate (Beats/min)**	84.33 ± 8.32	71.78 ± 12.93	**0.028**
**Systolic Blood Pressure (mmHg)**	110.00 ± 11.18	121.11 ± 9.28	**0.036**
**Diastolic Blood Pressure (mmHg)**	73.33 ± 5.00	76.67 ± 5.00	0.176
**VO**_**2**_**P**	21.89 ± 3.42	15.86 ± 4.34	**0.005**
**TC (mmol/L)**	4.98 ± 0.91	5.23 ± 0.94	0.112
**HDL (mmol/L)**	1.43 ± 0.48	1.21 ± 0.32	**0.001**
**LDL (mmol/L)**	3.08 ± 0.84	3.39 ± 0.89	**0.040**
**TG (mmol/L)**	0.93 ± 0.50	1.51 ± 1.00	**<0.001**
**FBG (mmol/L)**	5.11 ± 0.54	5.55 ± 1.04	**0.003**
**HbA1C (DCCT%)**	5.52 ± 0.41	5.83 ± 1.09	**0.040**
**ANGPTL8 (pg/ml)**	775.54 ± 46.12	1150.04 ± 108.10	**0.002**
**C-terminal 139–198 form of ANGPTL8 (ng/ml)**	0.20 ± 0.02	0.28 ± 0.04	**0.058**

### Full length and C-terminal 139–198 form of ANGPTL8 level in non-obese vs. obese subjects

**Fig 1:**
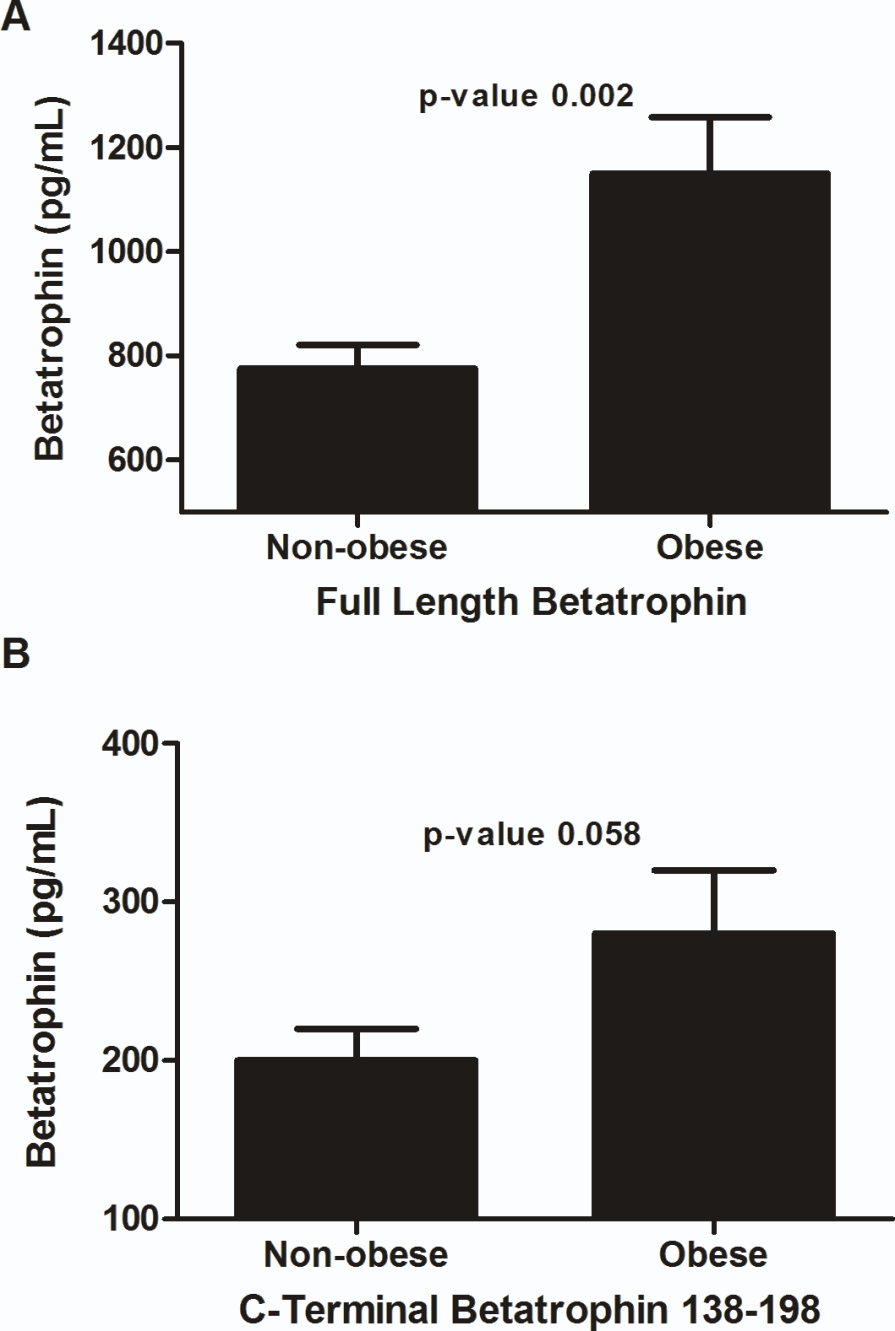
ANGPTL8 level obese vs. non-obese. **A**: Level of full length form of ANGPTL8 in non-obese vs. obese subjects in circulation. **B**: Level of C-terminal 139–198 form of ANGPTL8 in non-obese vs. obese subjects in circulation.

In this study, we have measured two forms of ANGPTL8 that have been suggested to exist in circulation (full length and C-terminal 139–198 form of ANGPTL8) and compared their level in non-obese vs. obese subjects. Full length ANGPTL8 was significantly higher in obese subjects compared to non-obese (775.54 ± 46.12 vs. 1150.04 ± 108.10 pg/ml, (*p*-value = 0.002)) Fig 1A. C-terminal 139–198 form of ANGPTL8 also showed significant increase in obese subjects relative to non-obese subjects with a p-value slightly higher than 0.05 (p-value = 0.058). Its level in non-obese was 0.20 ± 0.02 ng/ml compared to 0.28 ± 0.04 ng/ml in obese subjects Fig 1B.

### Clinical characteristics before and after exercise

Clinical characteristics of the obese subjects before and after exercise are shown in Table 2. Slight reduction was shown in BMI from 34.76 ± 3.22 to 33.36 ± 3.37 kg/m^2^ with a p-value of 0.099. Other measures such as percent body fat showed improvement after exercise, however, the difference did not reach significance (p-value = 0.127). VO_2_P on the other hand showed significant improvement from 15.86 ±4.34 to 22.42 ± 4.48 (p-Value = 0.011) in the obese subjects and slight improvement in the non-obese subjects from 21.41±3.56 to 23.87±5.41 (p-Value = 0.104). Non-obese subjects did not show any significant improvement in their anthropometric and clinical measure Table 2.

**Table 2:** Characteristics of the obese subjects before and after undertaking exercise training.

**Variable**	**Pre-Exercise Obese** Average ± SD	**Post-Exercise Obese** Average ± SD	**p-Value**
**BMI (kg/m**^**2**^**)**	34.76 ± 3.22	33.36 ± 3.37	0.099
**Percent Body Fat**	38.41 ± 5.76	36.04 ± 5.84	0.127
**Soft Lean Mass**	54.26 ± 11.64	54.80 ± 9.52	0.842
**Total Body water**	42.95 ± 8.93	43.27 ± 7.32	0.878
**Waist (cm)**	108.04 ± 10.83	105.21 ± 8.70	0.270
**Hip (cm)**	117.96 ± 8.87	114.10 ± 8.37	0.097
**Heart Rate (Beats/min)**	71.78 ± 12.93	80.88 ± 10.12	0.125
**Systolic Blood Pressure (mmHg)**	121.11 ± 9.28	120.00 ± 9.26	0.809
**Diastolic Blood Pressure (mmHg)**	76.67 ± 5.00	78.75 ± 3.54	0.334
**VO**_**2**_**P**	15.86 ± 4.34	22.42 ± 4.84	**0.011**
**TC (mmol/L)**	5.23 ± 0.94	5.11 ± 1.14	0.662
**HDL (mmol/L)**	1.21 ± 0.32	1.14 ± 0.25	0.334
**LDL (mmol/L)**	3.39 ± 0.89	3.35 ± 1.01	0.867
**TG (mmol/L)**	1.51 ± 1.00	1.44 ± 0.84	0.780
**FBG (mmol/L)**	5.55 ± 1.04	5.65 ± 1.06	0.715
**HBA1C (DCCT%)**	5.83 ± 1.09	5.85 ± 0.47	0.913
**ANGPTL8 (pg/ml)**	1150.04 ± 108.10	852.04 ± 51.95	**0.015**
C-terminal 139–198 ANGPTL8 (**ng/ml)**	0.28 ± 0.04	0.19 ± 0.03	**0.058**
**Variable**	**Pre-Exercise Non-Obese** Average ± SD	**Post-Exercise Non-Obese** Average ± SD	**p-Value**
**BMI (kg/m**^**2**^**)**	24.66 ± 2.86	25.21 ± 2.78	0.347
**Percent Body Fat**	31.58 ± 5.74	29.54 ± 5.07	0.095
**Soft Lean Mass**	42.78 ± 8.75	46.04 ± 9.89	0.131
**Total Body water**	33.59 ± 6.72	36.06 ± 7.63	0.136
**Waist (cm)**	85.38 ± 9.56	86.82 ± 10.74	0.539
**Hip (cm)**	103.71 ± 13.48	103.20 ± 6.49	0.068
**Heart Rate (Beats/min)**	84.33 ± 8.32	75.46 ± 13.62	0.073
**Systolic Blood Pressure (mmHg)**	110.00 ± 11.18	111.54 ± 6.89	0.720
**Diastolic Blood Pressure (mmHg)**	73.33 ± 5.00	74.62 ± 6.60	0.610
**VO**_**2**_**P**	21.41±3.56	23.87±5.41	0.104
**TC (mmol/L)**	4.98 ± 0.91	5.09 ± 0.74	0.234
**HDL (mmol/L)**	1.43 ± 0.48	1.55 ± 0.43	0.417
**LDL (mmol/L)**	3.08 ± 0.84	3.09 ± 0.93	0.587
**TG (mmol/L)**	1.29 ± 0.94	0.93 ± 0.50	0.062
**FBG (mmol/L)**	5.11 ± 0.54	5.01 ± 1.04	0.212
**HBA1C (DCCT%)**	5.52 ± 0.41	5.34 ± 0.60	0.351
**ANGPTL8 (pg/ml)**	775.54 ± 46.12	796.29 ± 40.55	0.736
C-terminal 139–198 ANGPTL8 **(ng/ml)**	0.20 ± 0.02	0.25 ± 0.05	0.382

### ANGPTL8 level before and after exercise

**Fig 2:**
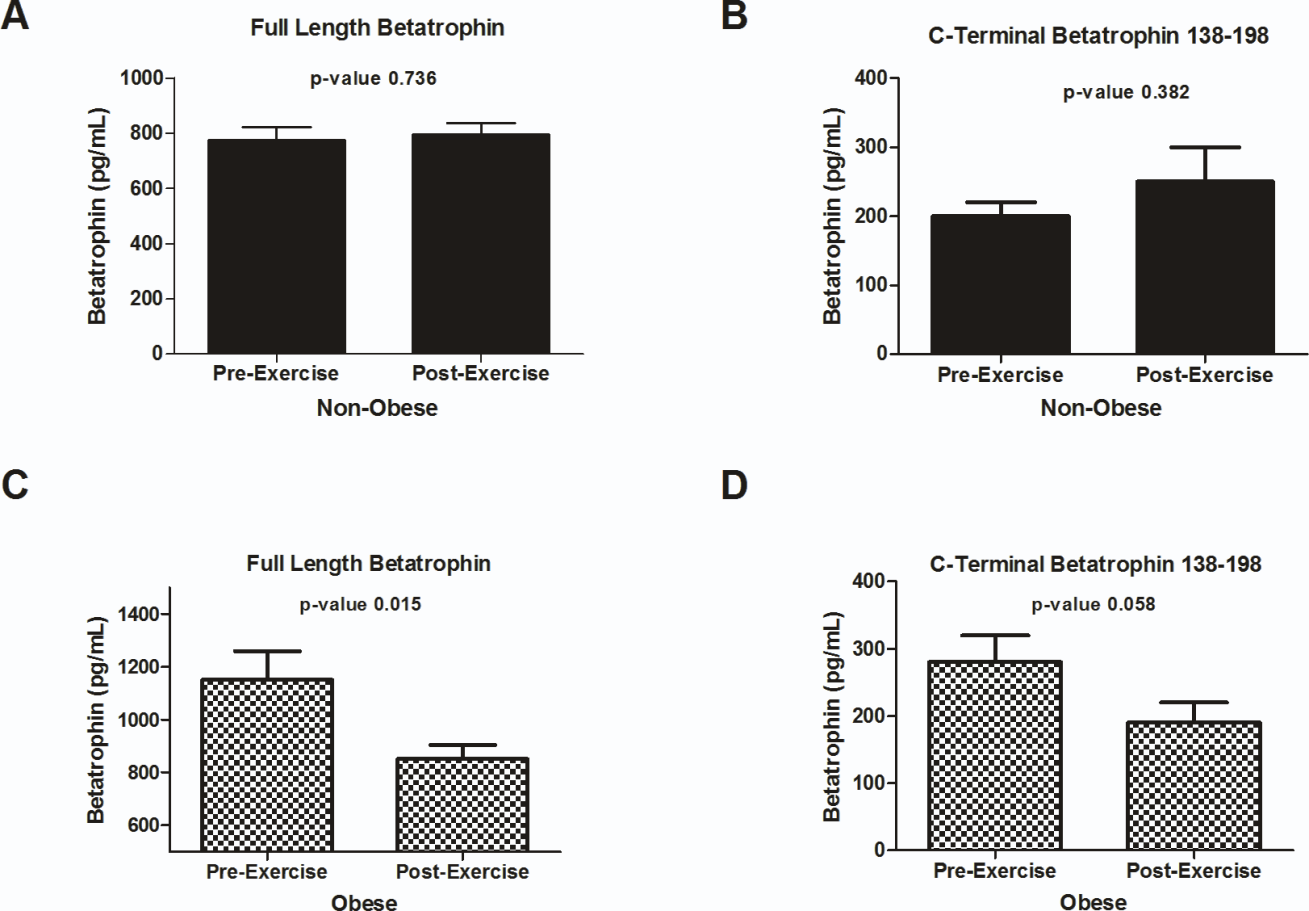
ANGPTL8 level after exercise. **A**: Level of full length form of ANGPTL8 in non-obese subjects before and after three months of exercise training. **B**: Level of C-terminal 139–198 form of ANGPTL8 in non-obese subjects before and after three months of exercise training. **C**: Level of full length form of ANGPTL8 in obese subjects before and after three months of exercise training. **D**: Level of C-terminal 139–198 form of ANGPTL8 in obese subjects before and after three months of exercise training.

The level of both forms of ANGPTL8 was assessed before and after exercise. Full length form of ANGPTL8 did not show significant changes in the non-obese subjects after the three months exercise (775.54 ± 46.12 vs. 796.29.04 ±40.55 pg/ml, (*p*-value = 0.736)) Fig 2A. Obese subjects on the other hand had a significantly lower level of full length ANGPTL8 after exercise. Its level was (1150.04 ± 108.10 pg/ml) before exercise and was reduced to (852.04 ± 51.95 pg/ml) after exercise (*p*-value = 0.015) Fig 2B. C-terminal 139–198 form of ANGPTL8 showed a similar trend where non-obese subjects did not have significant change in ANGPTL8 after exercise (0.20 ± 0.02 vs. 0.25 ± 0.05 ng/ml, (p-Value = 0.382)) Fig 2C. On the other hand, C-terminal 139–198 form of ANGPTL8 was lowered from (0.28 ± 0.04 ng/ml) before exercise to (0.19 ± 0.03 ng/ml) after exercise (p-value = 0.058) Fig 2D.

### Correlation between ANGPTL8 and FBG before and after exercise

**Fig 3:**
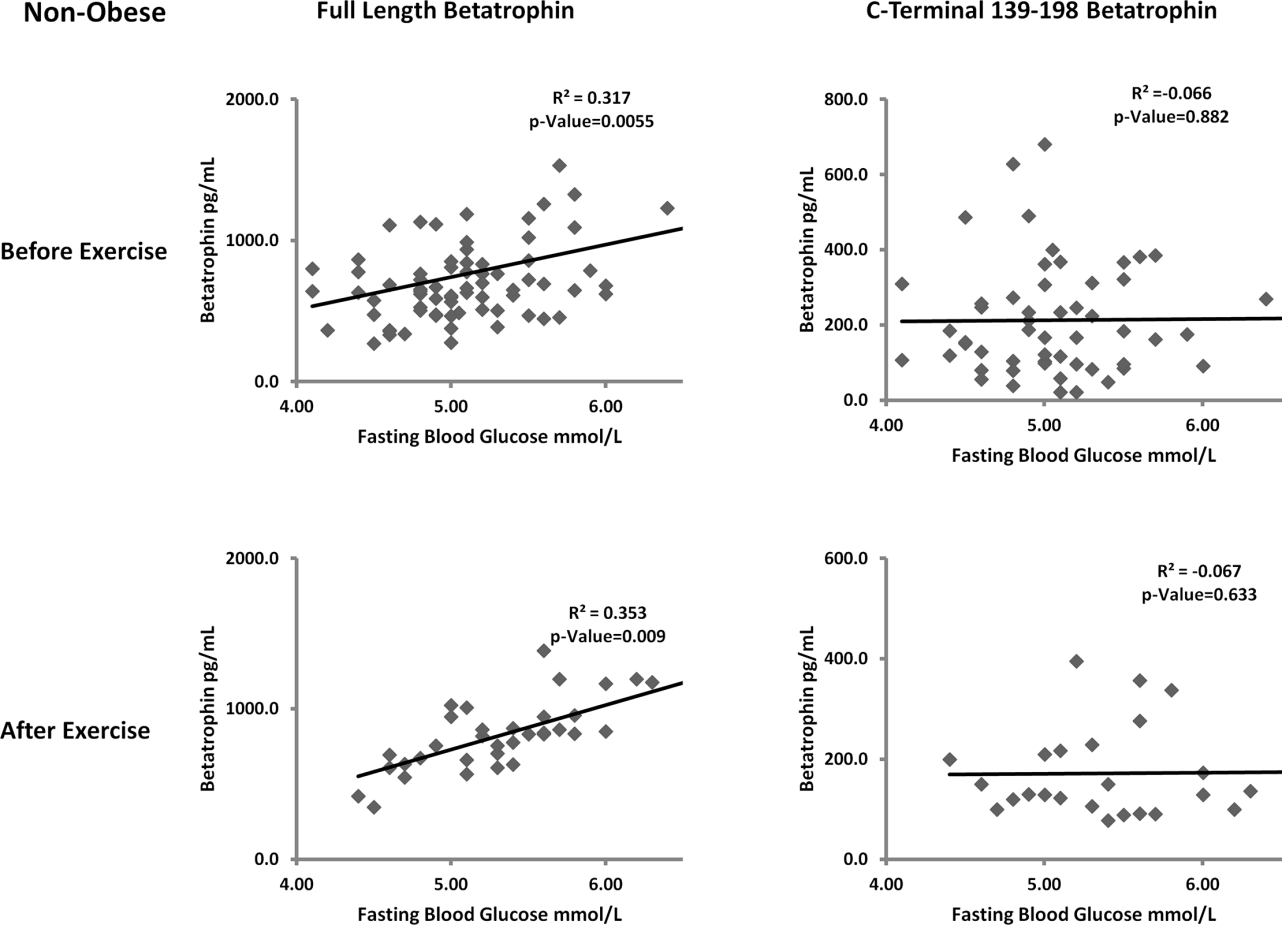
Spearman’s correlation between full length and C-terminal form of ANGPTL8 and FBG in non-obese subjects before and after exercise adjusted for age gender and BMI.

**Fig 4:**
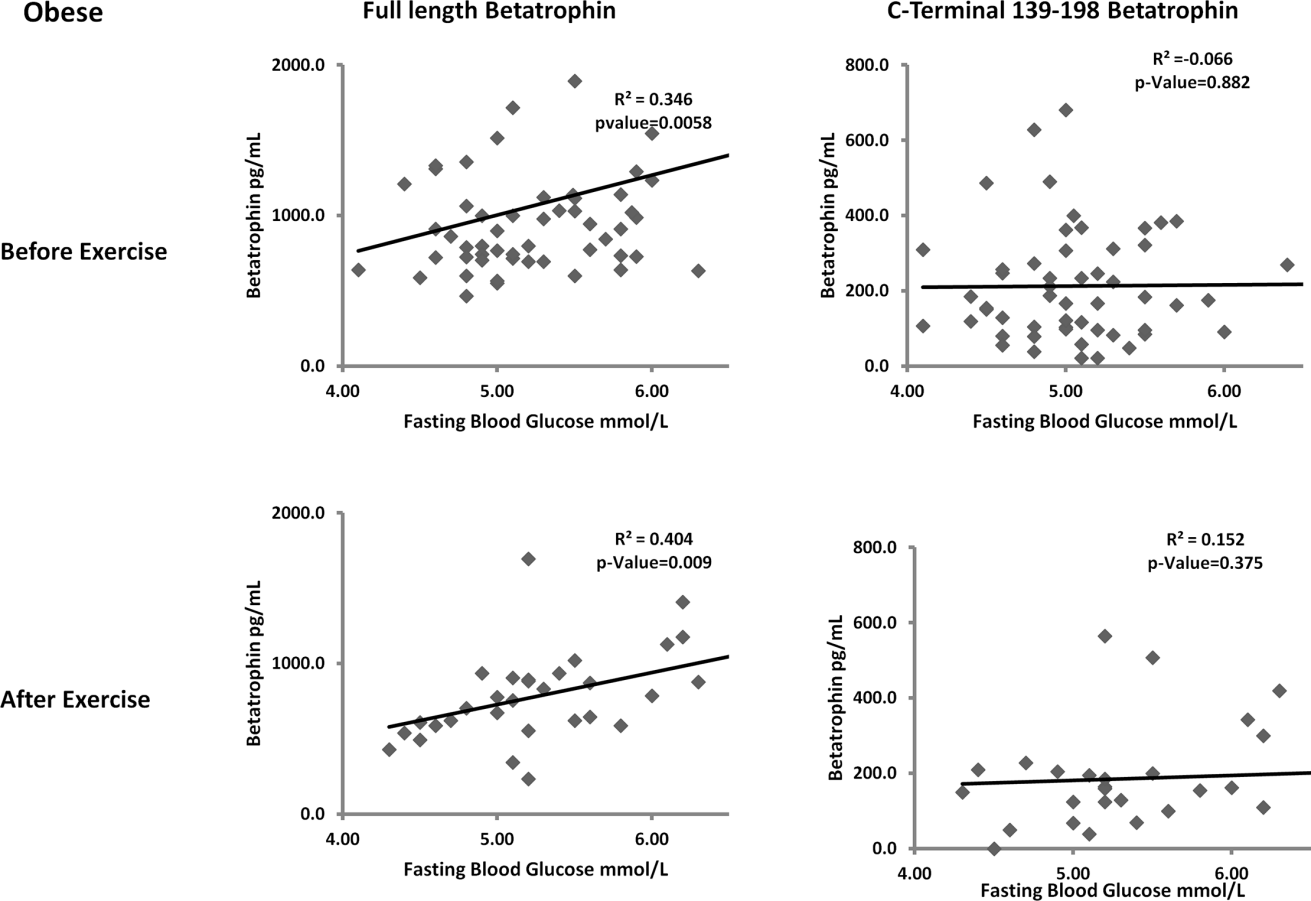
Spearman’s correlation between full length and C-terminal form of ANGPTL8 and FBG in the obese group before and after exercise adjusted for age gender and BMI.

In order to study the potential functional difference between the two forms of ANGPTL8, we looked at their association with FBG. We have previously shown that ANGPTL8 was positively associated with FBG in non-diabetic subjects. In this study, we showed that full length form of ANGPTL8 was positively correlated with FBG in the non-obese subjects before (r = 0.317, p-Value = 0.006) and after exercise (r = 0.353, p-Value = 0.035). The C-terminal 139–198 form of ANGPTL8 on the other hand did not show any significant correlation with FBG in the non-obese groups before and after exercise as shown in Fig 3. On the other hand, full length ANGPTL8 showed positive correlation with FBG in obese subjects before (r = 0.346, p-Value = 0.006) and after exercise (r = 0.404, p-Value = 0.009) Fig 4. No correlation was observed between the C-terminal 139–198 form of ANGPTL8 and FBG before and after exercise in the obese group as shown in Fig 4.

## Discussion

ANGPTL8 is a liver and adipose tissue produced protein that has been found to have a dual role in regulating triglyceride metabolism and glucose metabolism [5,8,9,10,11,29]. As a result ANGPTL8 has been suggested as a potential therapeutic target for dyslipidemia and diabetes [5,6,7,8,30]. Exciting yet conflicting data has emerged regarding the actual involvement of ANGPTL8 and its association with lipid and glucose homeostasis. In this study we attempted to answer some of these questions by studying the circulating level of two different forms of ANGPTL8 and their expression in obese and non-obese subjects before and after exercise as well as their association with blood glucose. Our data show that the levels of full length as well as C-terminal 139–198 forms of ANGPTL8 were increased in obese subjects and for the first time we showed that exercise intervention has led to reduction in the levels of both forms of ANGPTL8 in obese subjects. On the other hand, full length form of ANGPTL8 has shown significant correlation with FBG in both obese and non-obese subjects before and after exercise. However, the C-terminal 139–198 form of ANGPTL8 did not show any association with FBG before and after exercise intervention highlighting the need for full form of ANGPTL8 to modulate glucose homeostasis.

ANGPTL8 has also been given other names such as C19orf80, Hepatocellular Carcinoma-Associated Gene TD26 and RIFL (refeeding induced in fat and liver) [8,9,10,11]. ANGPTL8 has been shown to regulate the level of triglyceride in plasma through its interaction with another angiopoietin-like protein called ANGPTL3 [8]. Its overexpression was shown to cause hypertriglyceridemia while its inactivation led to reduction in TG level without affecting glucose level [8,30]. Its knockout in 3T3 L1 cells during adipogenesis and its knockdown led to a reduction in adipogenesis [9]. Due to its activity in lipoprotein lipase inhibition, ANGPTL8 was also called Lipasin; its expression was induced by cold shock at 4 ^0^C and high fat diet, while fasting reduced its expression [10,11]. ANGPTL8 has been implicated in a number of important metabolic related pathways such as lipid metabolism, insulin production and adipocytes differentiation. Initial studies suggested that ANGPTL8 was increased in obesity and knockdown of ANGPTL8 resulted in weight reduction in addition to reduction in TG level. Human data on the other hand were contradictory and showed a different expression pattern in obesity [21] [24]. Fu et al., 2014 has shown increase in the level of ANGPTL8 in obesity while [21] while Gomez-Ambrosi et al., 2014 showed reduction [24].We have recently showed that ANGPTL8 was increased in Type 2 diabetes and its level was correlating positively with obesity [15]. Since some of the discrepancies in measuring ANGPTL8 level has been suggested to be the result of using antibodies recognizing epitopes from the N- and C-terminal section of ANGPTL8, we used the two most commonly used ELISA kits EIAAB (catalogue number E1164H) and Phoenix Pharmaceuticals (catalogue number EK-051-55) to evaluate the level of ANGPTL8 from. In this study we measured both full length and C-terminal 139–198 form of ANGPTL8 and both show positive correlation with BMI. This data eliminates the discrepancy based on the kits used and suggest that the discrepancy in ANGPTL8 measurements might be due to ethnic variations or other unknown factors such as the protein degradation as suggested by Zhang et al. [31]. However, the reduction in ANGPTL8 level by exercise training independent of dramatic changes in BMI, as discussed below, further supports the involvement of other factors in the regulation of ANGPTL8.

Exercise training has been conclusively confirmed as a modulator and a potential treatment for obesity induced insulin resistance and diabetes [32,33]. The role of exercise in improving insulin sensitivity in obese subject is well studied through its ability to increase glucose uptake in muscles through enhancing the phosphorylation of Akt substrate of 160 kDa (AS160) [34]. In this study we demonstrated that ANGPTL8 level was decreased after exercise. It’s interesting that the reduction in ANGPTL8 level was only observed in obese subjects. Ren et al., 2012 has initially showed that ANGPTL8 null mice had a reduced fat mass, body weight and TG. They also showed that the transcript of ANGPTL8 (also called RIFL) was increased in obesity. Yi et al. also showed that ob/ob mice had a higher level of ANGPTL8 compared to lean. Quagliarini et al. highlighted the therapeutic potential of inhibiting ANGPTL8 in treating dyslipidemia since its overexpression was shown to increase triglyceride level while its knockout was shown to decrease it [8,30]. Collectively, these data support the notion that ANGPTL8 inhibition can be exploited as a potential therapeutic target especially after showing that exercise can reduce its expression in obese subjects.

The role of ANGPTL8 in beta cell proliferation has been recently challenged by Gusarova et al. suggesting that ANGPTL8 knockout did not affect beta-cell proliferation under insulin resistance state [12]. Nonetheless, in this study we show that ANGPTL8 is still correlating with FBG and potentially affecting glucose homeostasis in humans. We also show that the different forms of ANGPTL8 display different correlation with FBG as the C-terminal 139–198 form of ANGPTL8 did not show any correlation in both groups. This highlights the potential cause for the observed discrepancy in the published data [21,24] and the importance of measuring the full length form of ANGPTL8 while investigating glucose homeostasis. This result was also observed in an earlier study using another population consisting of over 1000 subjects where we showed strong positive correlation of the full length ANGPTL8 with FBG in non-diabetic subjects [15].

In conclusion, our data shows that both full length and C-terminal 139–198 form of ANGPTL8 were increased in obese subjects compared to non-obese subjects in our population. This increase in ANGPTL8 level was reduced in obese subjects that went through three months of exercise. We also show that full length ANGPTL8 was correlated positively with FBG in non-obese and obese subjects, while the C-terminal 139–198 form of ANGPTL8 did not show any correlation with FBG. Taken together, our data supports the potential therapeutic benefit of reducing ANGPTL8 level in obese subject and highlights the potential involvement of ANGPTL8 in glucose homeostasis in non-diabetic subjects.
